# Cx25 contributes to leukemia cell communication and chemosensitivity

**DOI:** 10.18632/oncotarget.5226

**Published:** 2015-08-19

**Authors:** Maksim Sinyuk, Alvaro G. Alvarado, Pavel Nesmiyanov, Jeremy Shaw, Erin E. Mulkearns-Hubert, Jennifer T. Eurich, James S. Hale, Anna Bogdanova, Masahiro Hitomi, Jaroslaw Maciejewski, Alex Y. Huang, Yogen Saunthararajah, Justin D. Lathia

**Affiliations:** ^1^ Department of Cellular and Molecular Medicine, Lerner Research Institute, Cleveland Clinic, Cleveland, OH, USA; ^2^ Department of Biological, Geological, and Environmental Sciences, Cleveland State University, Cleveland, OH, USA; ^3^ Department of Molecular Medicine, Lerner College of Medicine, Case Western University, Cleveland, OH, USA; ^4^ Department of Immunology and Allergy, Volgograd State Medical University, Volgograd, Russia; ^5^ Department of Translational Hematology and Oncology Research, Cleveland Clinic, Cleveland, OH, USA; ^6^ Case Comprehensive Cancer Center, Case Western University, Cleveland, OH, USA; ^7^ Department of Pediatrics, Case Western Reserve University, Cleveland, OH, USA

**Keywords:** leukemia, gap junctions, Connexin 25, cell-cell communication

## Abstract

Leukemia encompasses several hematological malignancies with shared phenotypes that include rapid proliferation, abnormal leukocyte self-renewal, and subsequent disruption of normal hematopoiesis. While communication between leukemia cells and the surrounding stroma supports tumor survival and expansion, the mechanisms underlying direct leukemia cell-cell communication and its contribution to tumor growth are undefined. Gap junctions are specialized intercellular connections composed of connexin proteins that allow free diffusion of small molecules and ions directly between the cytoplasm of adjacent cells. To characterize homotypic leukemia cell communication, we employed *in vitro* models for both acute myeloid leukemia (AML) and acute lymphoblastic leukemia (ALL) and measured gap junction function through dye transfer assays. Additionally, clinically relevant gap junction inhibitors, carbenoxolone (CBX) and 1-octanol, were utilized to uncouple the communicative capability of leukemia cells. Furthermore, a qRT-PCR screen revealed several connexins with higher expression in leukemia cells compared with normal hematopoietic stem cells. Cx25 was identified as a promising adjuvant therapeutic target, and Cx25 but not Cx43 reduction via RNA interference reduced intercellular communication and sensitized cells to chemotherapy. Taken together, our data demonstrate the presence of homotypic communication in leukemia through a Cx25-dependent gap junction mechanism that can be exploited for the development of anti-leukemia therapies.

## INTRODUCTION

Leukemia consists of a broad diagnostic category of hematological malignancies that are estimated to account for over 52,000 new cancer diagnoses in the United States, resulting in over 24,000 deaths annually [1]. Leukemia mainly affects the adult population, with a median age of 66 at diagnosis, but is also the most common cancer among children, accounting for almost 1 out of 3 pediatric cancers [1]. Additionally, while the term “leukemia” is used to describe the four major types of leukemia, acute lymphoblastic leukemia (ALL), acute myeloid leukemia (AML), chronic lymphocytic leukemia (CLL), and chronic myeloid leukemia (CML), these diseases share several defining features such as uncontrolled proliferation and self-renewal of immature lymphoblasts or myeloblasts that subsequently interfere with normal hematopoiesis. Clinically, patients present with symptoms such as anemia, neutropenia, and thrombocytopenia, which are dominant throughout treatment. The standard of care for leukemia includes chemotherapy, radiation, or both in combination often followed by autologous or allogenic stem cell transplantation [2]. While progress has been made in recent years toward understanding the underlying molecular mechanisms of leukemia, the average five-year survival rate is 57% in the United States and is largely dependent on the patient's age at diagnosis, gender, race, and type of leukemia [3].

The hematopoietic microenvironment is critical in the initiation and progression of leukemia [4]. The bone marrow niche comprises a heterogeneous population of hematopoietic and non-hematopoietic stromal cells as well as their extracellular products and cytokines. These cells produce a variety of different extracellular matrix (ECM) molecules including numerous interstitial and basal lamina collagens. In the close confines of the bone marrow, cell-cell and cell-ECM communication plays a large role in promoting hematopoietic progenitor cell (HPC) survival, expansion, and differentiation [5]. These interactions are also thought to be essential for the tumorigenesis and progression of leukemia. Recently, evidence has shown that direct contact of leukemia cells with the surrounding stroma inhibits their apoptosis and enhances their survival following chemotherapy [6]. Leukemia cells have also been shown to communicate with endothelial cells via exosomal miRNA [7] and may contribute to the angiogenic activity of endothelial cells, suggesting that cytoplasmic signals originating in one cell type are capable of entering another while impacting the phenotype in the recipient cell. However, there has been little evidence for the mechanism of direct homotypic communication between individual leukemia cells themselves and the implications this may cause for clinical treatment.

A common means for intercellular signals to quickly travel between two adjacent cells is through gap junction-mediated mechanisms. Gap junction communication is present in virtually all solid tissues and has been demonstrated to be essential for normal embryonic development [8], electric coupling in cardiac muscle [9], and neurons [10], and for normal hematopoiesis [11]. Additionally, connexin expression in non-excitable tissues has key roles in organ development [12], skeletal development and function [13], and growth control [14]. Gap junctions are defined as cell-cell junctions at which two plasma membranes from adjacent cells link. Gap junctions are specialized intercellular connections formed by a family of at least 24 human proteins called connexins that allow for the diffusion of small molecules and ions directly between the cytoplasm of adjoining cells [15]. Individual connexins show tissue-, cell-type-, or developmental stage-specific expression, and most organs as well as many cell types express more than one connexin. Six individual connexin proteins may co-oligomerize with the same (homomeric) or mixed (heteromeric) connexins into connexons, or hemichannels, although only certain combinations are possible [16]. Two hemichannels are then able to come together, forming homotypic or heterotypic gap junction channels between contacting cells, depending on connexon isotype, that facilitate cell-cell communication through the direct transfer of small molecules up to 1 kDa such as Ca^2+^, cyclic adenosine monophosphate (cAMP), and inositol triphosphate (IP_3_) [17].

The importance and variable nature of cell-cell communication in cancer has only recently begun to be carefully investigated, and as a result, current clinical models do not fully appreciate or take into account the effects this may have on the tumor phenotype. Given that many advanced cancers such as breast, prostate, and leukemia often present as histologically dense entities, it is reasonable to consider that cell-cell communication occurs and is able to confer a survival advantage to tumor cells. Additionally, while current therapies are effective at eradicating rapidly dividing cells, they have no way of distinguishing friend from foe, and many of the side-effects associated with treatment are the direct result of normal cell death. Therefore, the identification of tumor-specific gap junction function is paramount to develop effective, complimentary anti-tumor therapies aimed at removing malignant tissue while sparing normal healthy tissue. In the current study, we demonstrate that direct cell-cell communication between leukemia cells occurs, in part due to a Cx25-dependent mechanism that enables enhanced tumor cell proliferation and allows for the identification of additional potential specific gap junction protein functions as targets for anti-tumor therapy.

## RESULTS

### Leukemia cells are capable of direct communication

**Figure 1:**
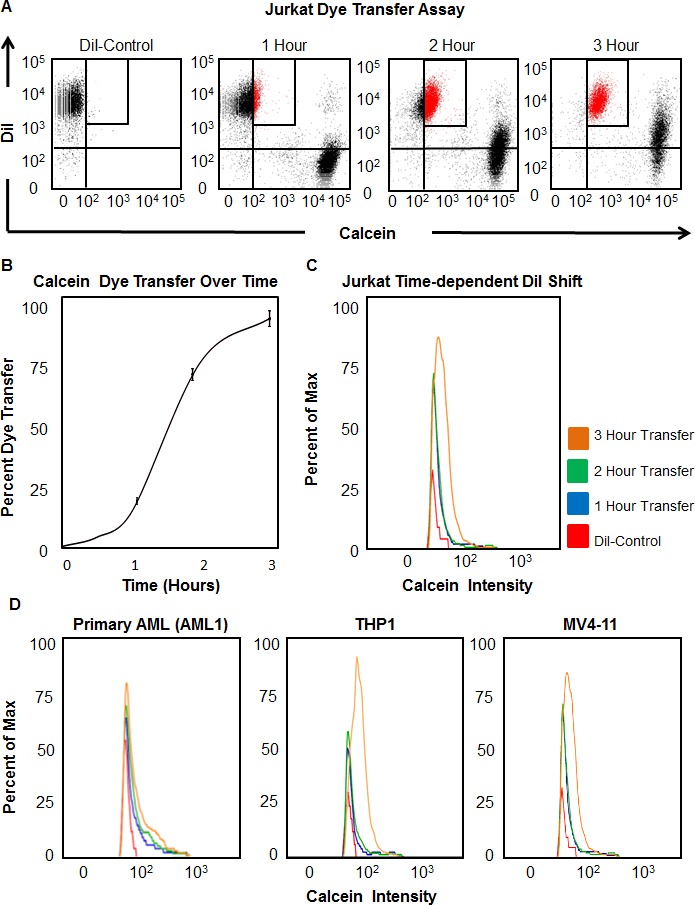
Functional gap junction activity is detectable directly between leukemia cells **A.** Jurkat cells were labeled with Calcein AM or DiI and then co-incubated for 1-3 hr. After incubation, cells were analyzed by flow cytometry. **B.** Quantification of the percent of dye transfer between leukemia cells as a function of time (1, 2, and 3 hr). **C.** Histogram of Calcein intensity in DiI-labeled Jurkat cells over time. **D.** Histogram of Calcein intensity in primary patient-derived AML cells, THP1 and MV4-11.

To assess whether direct cell-cell communication was present in leukemia cells, we used several cell lines to model both ALL and AML (Figure 1A–1D). After Calcein AM- and DiI-labeled Jurkat cells were mixed and incubated for 1 hr, flow cytometry analysis detected 4.1±0.4% Calcein transfer from Calcein-labeled cells to DiI-labeled cells (Figure 1B). Increasing the length of time that cells were allowed to mix permitted the Calcein to spread to more cells, indicative that dye transfer is a passive, on-going process. After 3 hr of co-incubation, dye transfer increased to 90.6±3.6%, confirming that connexin channels are functional in Jurkat cells (Figure 1B). The increase in Calcein transfer over time was further validated by the increase of fluorescence intensity over time (Figure 1C). This suggests that as cells interact with each other for increased lengths of time, Calcein transfers to a greater number of cells, thus amplifying the intensity of the signal. To better represent cell-cell communication in leukemia, dye transfer was also measured in a primary patient-derived AML cell line as well as in two characterized human AML cell lines, MV4-11 and THP1. The direct percentage of dye transfer was variable among all tested cell lines but confirmed that direct cell-cell communication is conserved among leukemia cells (Figure 1D).

Additionally, to test whether the detected dye transfer was mediated by direct physical contact between cells and was not a result of Calcein uptake from the cell culture media itself, we performed dye transfer assays with 0.4 μm Transwell inserts, thereby keeping the two groups of cells from making contact (Supplemental Figure 1A). After 1 and 3 hr of incubation, the control wells without inserts with physical cell-cell contact had a higher relative percentage of dye transfer than those that were separated by the Transwell inserts (Supplemental Figure 1B). In addition, to demonstrate that Calcein does not leak out of cells and into the surrounding media, flow cytometry was utilized to measure Calcein signal at 1, 2, and 3 hr. Indeed, leukemia cells did not lose intensity during the time course used for our experiments, arguing that dye uptake requires cell-cell contact. It should be noted that the percentage of transfer was highly variable among the four cell lines used (20-98% after 3 hr) and was dependent on several factors including cell type and the stochastic nature of cell-cell interactions *in vitro*, as we cannot control which individual cells form gap junction channels. However, taken together, these data demonstrate that functional and quantifiable cell-cell communication exists in leukemia and is dependent on physical cell-cell contact.

### Effects of gap junction inhibition on dye transfer

**Figure 2:**
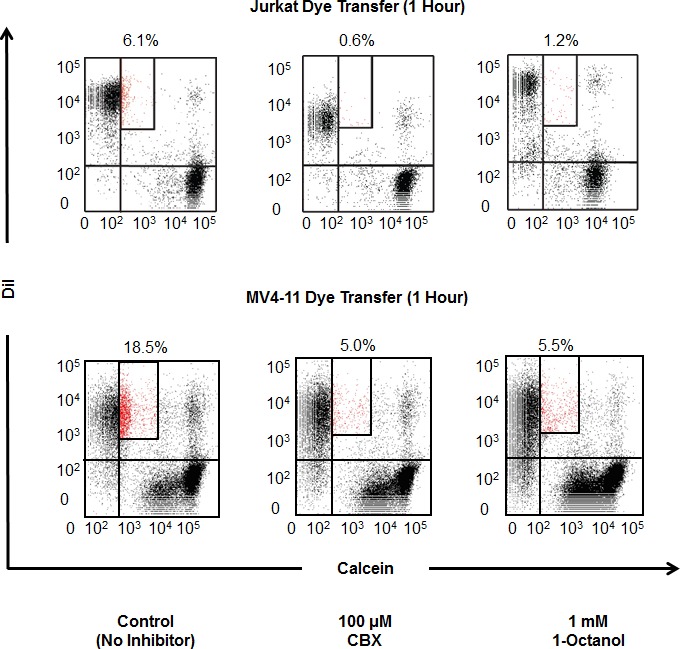
Pharmacological blockade of gap junctions is sufficient to attenuate communication between JURKAT and MV4-11 cells Gap junction activity was inhibited by two pan-gap junction inhibitors, CBX and 1-octanol, at pharmacologically relevant concentrations. After 1 hr of incubation, dye transfer was reduced in cells treated with the inhibitors.

To evaluate whether the observed cell-cell communication was gap junction dependent, two clinically relevant pan-gap junction inhibitors, CBX and 1-octanol, were used to inhibit gap junctions in leukemia cells. CBX is currently approved for the clinical treatment of esophageal and mouth ulcers in the United Kingdom [22], while 1-octanol is currently being investigated for the treatment of essential tremor [23]. Cells were stained with Calcein AM and DiI as previously described, treated with each inhibitor, and allowed to incubate for either 1 or 3 hr, after which the percent of Calcein dye transfer to DiI-labeled cells was measured using flow cytometry. After 1 hr of co-incubation, Jurkat cells showed a 6.1% transfer, with MV4-11 cells showing an 18.5% transfer. However, when co-incubated in the presence of 100 μM CBX or 1 mM 1-octanol, both Jurkat and MV4-11 cells exhibited reduced dye transfer percentages compared with their vehicle controls (Figure 2). Furthermore, after 3 hr of co-incubation with CBX and 1-octanol, Jurkat and THP1 cells continued to show a reduced percentage of dye transfer (Supplemental Figure 2), while MV4-11 cells did not demonstrate a reduction in dye transfer at this time point, indicating that slower compensatory communication mechanisms likely exist in leukemia cells when gap junctions are inhibited. These data demonstrate that homotypic cell-cell communication relies on functional gap junctions and may be blocked through pharmacological inhibition of connexin subunits.

### qRT-PCR screen of connexins in AML and JURKAT cells *versus* HSCs

**Figure 3:**
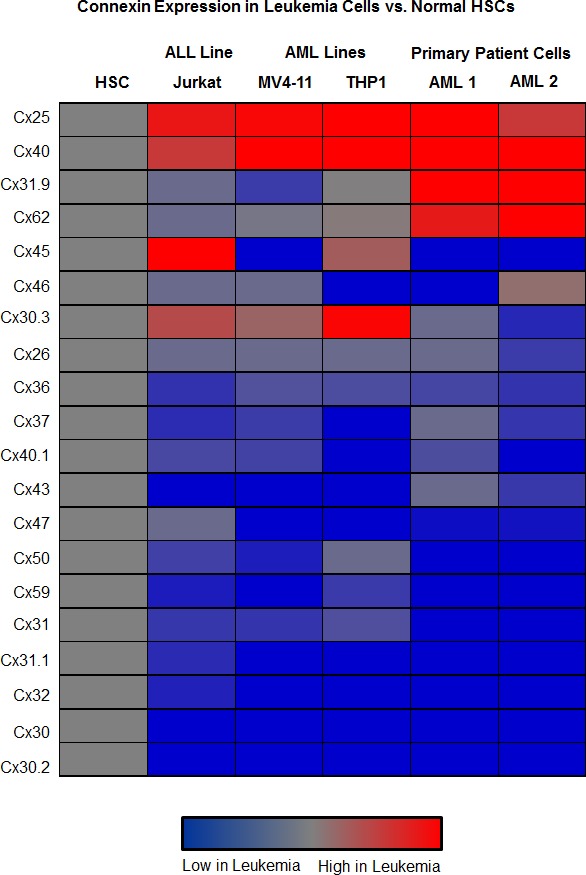
qRT-PCR analysis of connexin expression in leukemia mRNA profiles of connexin expression were interrogated in normal HSCs, Jurkat cells, two primary patient-derived AML cell specimens, and two AML cell lines, MV4-11 and THP1. Two connexins were found to be more highly expressed in all leukemia cells versus normal HSCs, Cx25 and Cx40, while primary AML cell lines expressed higher levels of Cx31.9 compared with HSCs.

To identify whether specific connexins are expressed in leukemia, a qRT-PCR screen of known connexin subunits was employed. Normal hematopoietic stem cells (HSCs) were probed to identify tumor-specific connexins important in leukemia cells but not healthy controls. Three connexins were found to be increased in all leukemia cell lines tested: Cx25, Cx40, and Cx31.9 (Figure 3). Bioinformatics data using RNA-seq were subsequently generated to narrow down those connexins that were detected in the Cancer Genome Atlas AML dataset [20]. Samples were organized by the French American British (FAB) morphological categories, with the group expressing high Cx25/GJB7 consisting of M3 AML. Consequently, Cx25 and Cx31.9 were found to be expressed in both the qRT-PCR screen as well as by bioinformatics (Supplemental Figure 3).

### Cx25 knockdown inhibits leukemia cell-cell communication

**Figure 4:**
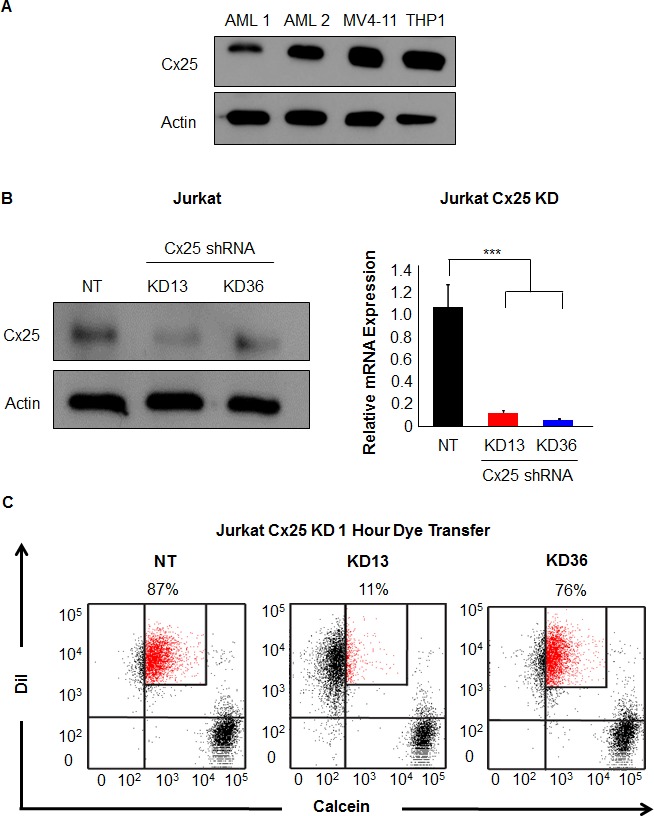
Targeting Cx25 by RNAi decreases cell-cell communication **A.** Immunoblot analysis of Jurkat cells, two primary patient-derived AML cell lines, and two AML cell lines to assess Cx25 protein expression. **B.** Inhibition of Cx25 by shRNA-mediated knockdown showed decreased protein expression following transduction with two targeting constructs. qRT-PCR was utilized to validate the shRNA constructs and determined that both KD13 and KD36 were able to decrease Cx25 mRNA expression. **C.** Following inhibition of Cx25, Calcein dye transfer was reduced in Jurkat cells at 1 hr compared with the NT control.

By PCR-based analysis, Cx25 and Cx40 were identified as potential tumor-promoting connexin subunits expressed in both primary AML cells and Jurkat cells, while Cx31.9 was expressed by primary AML cell lines. To validate our observation at the protein level, immunoblot analysis of Cx25 and Cx31.9 was utilized. Cx25 protein expression was found in all leukemia cell lines tested (Figure 4A, Supplemental Figure 4A), although Cx31.9 protein expression was undetectable (data not shown). In addition, Cx25 expression was visualized in both Jurkat and THP1 cells using immunofluorescence as both Jurkat and THP1 cells were found to express Cx25 on cell membranes (Supplemental Figure 4C). To further confirm the role of Cx25 in leukemia, we utilized a genetic approach to disrupt Cx25 by RNA interference (RNAi). We obtained two independent short hairpin RNA (shRNA) constructs to knock down Cx25 expression (knockdown 13 (KD 13) and knockdown 36 (KD 36)) in Jurkat cells. Compared with a nontargeting (NT) control, both Cx25 knockdown constructs reduced Cx25 expression as evaluated by immunoblotting and qRT-PCR (Figure 4B). Dye transfer assays were subsequently utilized to measure whether cell-cell communication was disrupted after Cx25 knockdown. A decrease in dye transfer was observed in Cx25 knockdown cells after 1 hr of incubation (11% dye transfer in KD 13 cells and 76% dye transfer in KD 36 cells) versus the NT control (87% dye transfer) (Figure 4C). However, after 3 hr of incubation, the percent of transfer was similar in all three groups, indicating the presence of additional compensatory communication mechanisms not dependent on Cx25.

### Cx25 knockdown sensitizes leukemia cells to chemotherapy

**Figure 5:**
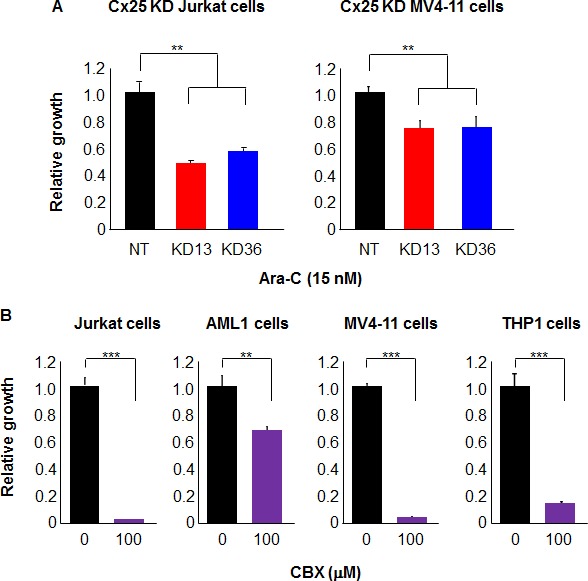
Cx25 KD increases leukemia cell chemosensitivity, while treatment with a gap junction inhibitor decreases leukemia cell proliferation **A.** Treatment of Cx25-knockdown Jurkat and MV4-11 cells with 15 nM Ara-C reduced their proliferative activity. **B.** Inhibiting gap junction activity in Jurkat cells, one primary patient-derived AML specimen, and two AML cell lines with 100 μM CBX reduced cell proliferation.

Following Cx25 knockdown, the proliferative capability of leukemia cells was interrogated but did not show a reduction compared with NT controls (Supplemental Figure 4B), indicating that the disruption of one connexin subunit was not sufficient to induce apoptosis. Interestingly, when Cx25 knockdown cells were incubated in the presence of the chemotherapeutic agent Ara-C at a concentration much lower than previously reported in the literature [24], the knockdown cells demonstrated a reduced capability to proliferate compared with their NT counterparts when incubated with 15 nM Ara-C (Figure 5A), suggesting that gap junction inhibition in combination with chemotherapy may be a potentially viable treatment strategy. These results indicate that Cx25 knockdown sensitizes leukemia cells to chemotherapeutics *in vitro* and may justify gap junction inhibition as an addition to current standard-of-care regimens. Although Jurkat cell proliferation was not affected by Cx25 knockdown, the disruption of cell-cell communication suggests a therapeutic potential for the targeting of gap junction function in leukemia. To that end, we further interrogated the phenotype of Cx25 knockdown in Jurkat and MV4-11 cells. We hypothesized that inhibition of Cx25 may sensitize leukemia cells to chemotherapeutics, as they would respond less rapidly to cellular damage. Indeed, when Cx25 was knocked down in Jurkat and MV4-11 cells, both constructs decreased proliferation in the presence of 15 nM Ara-C (also known as arabinofuranosyl cytidine), a common chemotherapeutic agent used for the treatment of AML and non-Hodgkin lymphoma (Figure 5A). To test whether this phenomenon was specific for Cx25, we similarly knocked down Cx43 with two shRNA constructs previously described in prostate cancer [19]. The decision to focus on Cx43 as a control rather than the other connexin subunits identified in the qRT-PCR screen was mediated by the fact that Cx43 is heavily studied in a wide variety of normal and pathophysiological contexts and would allow for a phenotypic comparison following shRNA disruption of either subunit. After confirming Cx43 knockdown with qRT-PCR (Supplemental Figure 5A), we treated Jurkat cells with 15 nM Ara-C and found that Cx43 inhibition did not sensitize the cells to chemotherapy (Supplemental Figure 5B). In addition, we performed dye transfer assays with Cx43-knockdown Jurkat cells and found that dye transfer was not affected at any time point (Supplemental Figure 6), indicating that Cx25 rather than Cx43 plays an important role in leukemia cell communication and chemosensitivity. Finally, we wanted to elucidate whether pharmacological inhibition of connexins is a feasible strategy for the development of future therapeutics. All four cell lines tested showed a decreased rate of proliferation following treatment with 100 μM CBX (Figure 5B). However, 1-octanol was less effective at decreasing proliferation, as treatment with several concentrations did not affect Jurkat cell growth (Supplemental Figure 7). These data demonstrate that pan-gap junction inhibition has a negative effect on leukemia cell growth, while disrupting Cx25 is sufficient to chemosensitize both Jurkat and MV4-11 cells.

### Cx25 is highly expressed in leukemia cell lines compared with other tumors

**Figure 6:**
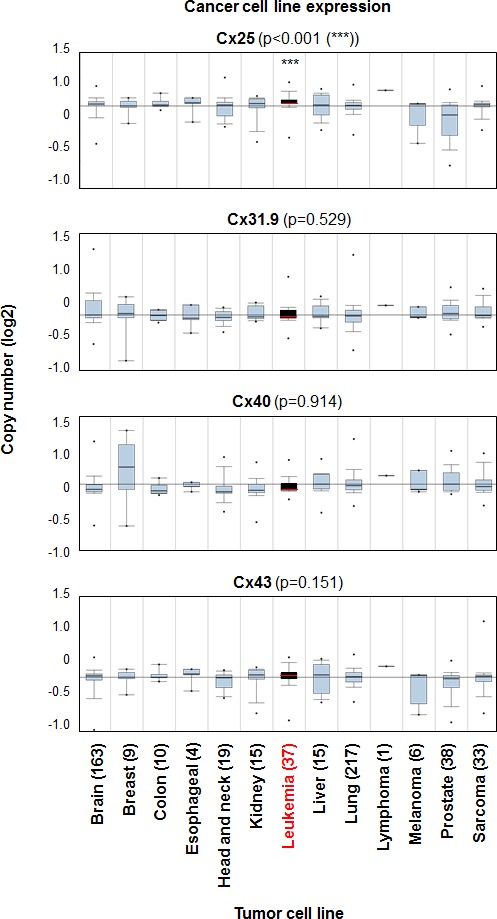
Cx25 expression is elevated in leukemia cell lines compared with additional tumor cell lines and connexin subunits Box and whisker plots of connexin subunit expression across multiple cancer cell lines demonstrates that Cx25 is significantly elevated in leukemia cell lines (black box) compared with other tumor types. Data accessed from the Beroukhim et al., Nature 2010 dataset [21] in Oncomine and number of tumor cell lines per groups is indicated for each tumor. Boxes span 25th-75^th^ percentile, line represents median value, bars represent expression range, and *** *p* < 0.001.

We have shown *in vitro* that inhibition of Cx25 can sensitize leukemia cells to chemotherapy; however, there is limited information as to the role of Cx25 in leukemia *in vivo.* We interrogated Cx25 expression across multiple tumor cell lines and found significantly higher expression in leukemia cell lines as compared with other tumors (Figure 6). This was not observed with other highly expressed connexins identified in Figure 3 (Cx40 and Cx31.9) and Cx43. Taken together, these data demonstrate that Cx25 may have a unique role in leukemia cell communication and may serve as an attractive target for the development of future adjuvant therapeutics.

## DISCUSSION

The cellular microenvironment plays a major role in tumorigenesis by providing nutrients and survival signals to cancer cells [25]. Additionally, the microenvironment may protect tumor cells from normal immune responses and promote resistance to therapeutic treatment regimens. Evidence in ALL has demonstrated that osteoblasts secrete an ECM molecule, osteopontin (OPN), that plays an important role in anchoring leukemia blasts in anatomic microenvironmental locations that support tumor cell dormancy and protect them from cytotoxic chemotherapy [26]. In other cases, tumor cells are even able to co-opt their microenvironment by crowding out normal cells, subsequently fashioning their milieu to provide them with a competitive survival advantage [4]. However, in many instances, the direct mechanisms by which tumor cells utilize their surroundings is poorly understood. While tumor cells may secrete various stimulatory growth factors and cytokines such as TNF-α, IL-6, TGF-β, and IL-10 to avoid immunosurveillance and create a pro-tumorigenic environment, little is yet understood about how direct cell-cell communication between the tumor cells, rather than tumor-stroma interactions, influences malignancy.

Here, we have shown that leukemia cells directly communicate with each other through at least one specific connexin, Cx25, which is increased in primary patient AML cells compared with normal hematopoietic cells. Likewise, Cx25 expression was also detected in Jurkat cells, a model for ALL, as well as in MV4-11 and THP1 cells, both models for AML. Using RNAi strategies to disrupt Cx25 in Jurkat and MV4-11 cells, we found that the communicative ability of the cells was reduced, which translated to increased chemosensitivity following treatment with Ara-C. This finding is clinically relevant, as one of the major challenges in the treatment of leukemia is the tolerability of current anti-leukemia agents. Children, the elderly, and those in general poor health are particularly susceptible to the side-effects of chemotherapy and are often unable to be treated. Future studies should be aimed at determining if Cx25 is linked to leukemia patient survival across multiple leukemia subtypes. However, this may be challenging as many databases do not contain Cx25 and these data may need to be generated. To further translate our findings, leukemia cell were treated with clinically relevant gap junction inhibitors, CBX and 1-octanol, at pharmacologically relevant concentrations [22, 23], and cell-cell transfer of Calcein decreased. However, only CBX was found to be effective at reducing leukemia cell proliferation. The inhibition of gap junction communication in leukemia cells followed by treatment with chemotherapeutics may allow for a much lower chemotherapy dose to be used, achieving the desired results while sparing patients from the harmful effects inherent to cancer treatment. However additional *in vitro* and *in vivo* studies are critical to determine the exact mechanisms behind gap junction-mediated cellular communication in leukemia. A wider screen of chemotherapeutics would also be necessary to investigate which combinatorial adjuvants best complement each other in pre-clinical models of leukemia.

Physical contact between cells was also found to be necessary for functional gap junction activity, as prohibiting this interaction did not allow for Calcein transfer. The ability of leukemia cells to communicate with each other and their surrounding microenvironment through gap junctions has the potential to affect the malignancy of leukemia. Cells are better able to respond to external stimuli and escape damage from sources such as chemotherapeutics and radiation by exchanging information amongst themselves. In addition, gap junctions may allow for the release of potentially lethal intercellular components such as reactive oxygen species (ROS) in response to cell damage or may facilitate the uptake of molecules that protect from ROS-induced DNA damage [27]. Recent work on gap junctions in HSCs has confirmed that Cx43-deficient HSCs and HPCs displayed decreased survival and increased senescence mediated by their inability to transfer ROS to the hematopoietic microenvironment following myeloablation [28]. These results demonstrate that Cx43 plays a protective role during stressful conditions such as hematopoietic recovery.

Targeting gap junction-mediated communication in leukemia and other cancers is emerging as an exciting prospective strategy with easily translatable results [29,19]. Specifically, the additive survival advantage that gap junction inhibition in combination with chemotherapy confers in animal models of glioblastoma is particularly promising [30]. However, several caveats remain to be addressed regarding both CBX and 1-octanol before clinical trials are implemented. Both agents demonstrate remarkable efficacy for inhibiting gap junctions and tumor cell growth in vitro and in vivo. However, their mechanism of action is poorly understood. In particular, it should be noted that these compounds do not specifically block individual connexin subunits or gap junctions. Rather, they are pan-gap junction inhibitors, ostensibly blocking the function of all connexins and making it difficult to study the particular connexin subunits that are involved in tumor biology. It is also important to remember that these inhibitors are capable of blocking additional hemichannels, such as pannexins, necessating careful examination of the molecular signaling pathways behind the mechanisms of CBX and 1-octanol function, as current reports remain contradictory. Less is known about connexin hemichannel activity, although both CBX and 1-octanol have been shown to be capable of disrupting hemichannels in keratinocytes [31] and spinal cord astrocytes [32], inhibiting ATP and chemokine release, respectively. Functional connexin and pannexin hemichannels have also been described to play a role in leukocyte adhesion [33], as demonstrated by decreased adhesion to venular endothelium after treatment with CBX. However, a contradictory study reported that reducing Cx40 levels instead increased leukocyte adhesion to mouse endothelial cells [34]. These contrasting conclusions are likely a result of the tissue-specific function of connexins *in vivo* and highlight the complexity of connexin function. It is also important to note that blocking all gap junctions may have unintended off-target effects that need to be addressed before considering clinical trials. Additionally, the exact method by which the agents inhibit connexin function is an ongoing area of investigation. It has been hypothesized that both CBX and 1-octanol act on cell membranes to alter fluidity and disrupt the transmembrane domains of connexin proteins, rendering them inert. However, this explanation has yet to be fully investigated and remains speculative.

The ablation of highly expressed connexins is not sufficient to target and destroy leukemia cells, nor is it sufficient to completely prevent cell-cell communication. Targeting specific connexins may also have secondary effects or cause a concomitant increase in the expression of other connexin proteins, leading to unintended phenotypic consequences. It is prudent to consider that connexins may possess additional functions that have yet to be fully described. To this end, cytoplasmic partners capable of interacting with the intracellular domains of connexin proteins may provide a potential means of specifically targeting individual subunits. The ablation of one universal connexin may have unintended secondary effects or no effects at all, as compensatory mechanisms likely exist among various connexin proteins. Rather, gap junction inhibition strategies should be contextualized in light of the overall tumor or, even more effectively, in light of the cell-of-origin of the tumor, to target the root of the malignancy rather than the branches. Of paramount importance is the development of novel mimetic peptides or agents capable of disrupting individual connexin subunits to minimize the harm to normal tissue in the course of treatment. Cancer therapy as a whole is moving away from a “one-size-fits-all” paradigm and toward a more individualized model. Targeting specific connexin subunits, depending on tumor subtype, is therefore complementary to the emerging trends regarding cancer care and should be considered for further attention.

Following radiation therapy, gap junctions also play an important role in the “bystander effect,” in which cells that are not directly exposed to radiation but are in the vicinity also respond to the exposure and display increased levels of genetic change and lethality. Recent work using genetic approaches to downregulate Cx43 demonstrated that gap junction-mediated communication is crucial for the transmission of radiation, causing tumor responses in the distal CNS in areas not exposed to direct radiation therapy. Surprisingly, Cx43 was also found to be upregulated in nontargeted tissue following irradiation, which may allow for the transduction of potentially oncogenic signals to remote tissue through the bystander effect [35]. An attractive additional strategy for radiotherapy would be the identification of tumor-specific gap junctions through which “death signals” may be transferred into the extracellular microenvironment to affect adjacent tumor cells that may have been protected from radiation. Alternatively, tumor-specific antigens may be released through gap junctions after radiotherapy or chemotherapy, priming the innate immune response to identify and eradicate unaffected tumor cells. This underlines the need to study gap junctions in the context of cancer and develop novel connexin inhibitors for pharmacological use [36].

Additional work is needed to determine the molecular mechanisms of gap junction signaling to gain a better understanding of the downstream signaling components behind connexin activation or inhibition. We have taken the first steps toward characterizing connexin function in leukemia, although many hurdles remain before gap junction inhibition will become an important clinical tool for cancer therapy. However, our results are an important proof-of-principle example, demonstrating the value in exploring and exploiting novel concepts in the fight against cancer.

## MATERIALS AND METHODS

### Cell culture and preparation of culture medium

Two different *in vitro* systems modeling ALL (Jurkat cells) and AML (THP1 and MV4-11) along with two primary patient-derived AML specimens (AML1 and AML2) were utilized to study gap junction communication in leukemia. Jurkat cells were cultured in RPMI 1640 supplemented with 10% heat-inactivated fetal bovine serum (FBS; Sigma), 2 mM sodium pyruvate, 5×10^−5^ M 2-mercaptoethanol, penicillin, streptomycin, L-glutamine, and 0.1 mM non-essential amino acids at 37°C in a humidified atmosphere of 20% oxygen and 5% CO_2_. The primary AML cells used in this study (AML1, AML2) have previously been described [18] and were obtained from patients with relapsed/refractory AML according to approved Cleveland Clinic IRB protocols. These cells had a myelomonocytic morphology (M4) and multiple chromosomal abnormalities including t(8;18)(q22:q23) and t(11;13)(q21:q12). Primary AML cells were cultured in IMDM supplemented with 10% FBS and 10 ng/ml of the following human cytokines: stem cell factor (SCF), FLT3 ligand, thrombopoietin, interleukin-3 (IL-3), and interleukin-6 (IL-6) (Life Technologies). The THP1 and MV4-11 cell lines were cultured in RPMI supplemented with 10% heat-inactivated fetal bovine serum (FBS; Sigma), penicillin, streptomycin, and L-glutamine.

### Isolation of CD34+ HSCs

CD34+ cells from bone marrow aspirates were immunopositively purified using a magnetic cell sorting system (Miltenyi Biotec) per the manufacturer's instructions and used as a negative control. Briefly, bone marrow aspirates were gathered and frozen in 10% dimethyl sulfoxide (DMSO) and FBS. Cells were stored in liquid nitrogen until needed. After thawing, cells were purified using a magnetic cell sorting system (CD34 MicroBead Kit #130-046-702, Miltenyl Biotec Inc., Auburn, CA, USA) according to the manufacturer's instructions. CD34+ cells were considered normal HSCs and were cultured in IMDM supplemented with 10% fetal bovine serum and 10 ng/ml of the following human cytokines: stem cell factor, FLT3 ligand, thrombopoietin, IL-3, and IL-6.

### Dye transfer assay

For dye transfer assessments, cells were divided into two groups. One group was labeled with 1 mM Calcein AM (Life Technologies), a cell-permeable dye in its parent form that is converted to the green-fluorescent Calcein after acetoxymethyl ester hydrolysis by intracellular esterases, after which it is no longer cell permeable and only able to leave the cell via gap junctions, hemichannels, or exocytosis. The other group of cells was labeled with 3 μM DiI (Life Technologies), a lipophilic membrane stain that diffuses laterally to stain the entire cell and does not leave the cell membrane. In both cases, cells were labeled for 45 minutes with either Calcein AM or DiI according to the manufacturer's instructions before centrifugation to remove excess dye. These two groups of cells were then mixed at a 1:1 ratio; incubated for 1, 2, or 3 hr; and analyzed by flow cytometry using a BD Fortessa to quantify the percentage of transfer of green calcein to DiI-labeled cells. Single DiI- and Calcein-stained control cells were used to verify the efficacy of the dyes and to set flow cytometry gates. After functional gap junction activity was confirmed, cells were incubated in the same manner as above in the presence or absence of the gap junction inhibitors carbenoxolone (CBX) and 1-octanol and likewise analyzed using flow cytometry. Flow cytometry experiments analyzed four different biological samples (Jurkat cells, one primary patient-derived AML specimen, and two different AML lines, MV4-11 and THP1). For the dye transfer assay, 20, 000 events were collected from a total of five groups of 1.0×10^6^ cells of each biological sample (one vehicle control and two carbenoxolone and two 1-octanol concentrations).

### Transwell assay

To confirm that dye transfer required direct cell-cell contact, cells were divided into two equal groups. One group was pre-loaded with Calcein AM while the other was pre-loaded with DiI.

Cells were subsequently incubated for 1, 2, or 3 hr in 6-well dishes with 12 mm Transwell Inserts containing a 0.4 μM pore (Corning) used to keep the two groups physically separated (Schematic provided in Supplemental Figure 1A). While in culture, the Calcein group was incubated in the apical chamber of the insert to ensure that if dye was released into the media, the donor DiI-labeled cells in the basolateral chamber would be capable of taking it up and subsequently fluorescing green. After incubation, both cell populations were mixed, and dye transfer was quantified using flow cytometry as described above.

### Proliferation assay

To quantify leukemia cell proliferation, a CellTiter-Glo^TM^ proliferation assay (Promega) was utilized. Briefly, cells were plated in clear-bottom 96-well plates at a density of 1000 cells per well in 100 μL of culture media. In addition, to test whether Cx25 knockdown was capable of sensitizing leukemia cells to chemotherapy, cells were incubated in the presence or absence of arabinofuranosyl cytidine (Ara-C), also known as cytarabine, and proliferation was measured at Day 0 and Day 3. Cells were allowed to recover for at least 2 hr before the first measurement was taken, which served as a loading control. The luminescence intensity of each well was measured using a luminometer (PerkinElmer) at several time points after plating. Each sample was run in triplicate to evaluate variablility. To prepare relative growth curves, the data were normalized to day 0 to account for any variation during preparation.

### Quantitative real-time PCR

Total RNA was isolated using TRIzol (Life Technologies) according to the manufacturer's instructions. cDNA synthesis and amplification from 1 μg mRNA via PCR was performed using the qScript^TM^ cDNA Supermix (Quanta Biosciences) with an Eppendorf Vapo Protect Mastercycler Pro (Eppendorf). This kit contains both oligo(dT) and random primers. Gene expression was measured by real-time PCR using the RT^2^ SYBR Green ROX^TM^ qPCR Mastermix Kit (SABiosciences). Connexin primers were utilized for 20 different connexins as previously described [19] and validated by verification of single-peak melt curves. The PCR product for Cx25 was further validated by the verification of a single band on an agarose gel. The PCR reaction and detection were performed with the ABI 7000 Sequence Detection System (Applied Biosystems). To minimize variability due to single housekeeping gene adjustment, individual connexin expression levels were normalized to multiple housekeeping genes (β-actin and GAPDH). After adjustment to housekeeping genes, the difference in cycle numbers for each individual connexin was further normalized to that of HSCs. The fold change was subsequently calculated by squaring the cycle difference between each tumor cell type and HSCs, and each technical replicate was performed in triplicate. The cycle number for each connexin subunit after adjustment to housekeeping genes is provided in Supplemental Table 1.

### Detection of Cx25 by immunoblotting

Immunoblot analysis was performed on the whole-cell extracts of primary AML specimens, Jurkat cells, and primary GBM cells used as a control. Cells were lysed in 10 mM Tris HCl, pH 7.4; 0.5% IGEPAL CA-630 (weight/volume); 150 mM NaCl; 1 mM EDTA; 2 mM sodium orthovanadate; 1 mM PMSF; and a 1:100 dilution of protease inhibitor cocktail for mammalian cells (P8340 Sigma), followed by protein determination using a Pierce BCA Protein Assay Kit (Thermo Scientific). Protein (20 μg) from cells was separated by SDS-PAGE using a 12% gel and then transferred to a PVDF membrane. After blocking the membrane with 5% milk in TBST, total Cx25 was detected using rabbit polyclonal anti-Cx25 (Sigma, SAB4501629), then incubated with the secondary antibody linked to horseradish peroxidase. The immunoreactive bands were visualized by Pierce ECL 2 Western Blotting Substrate (Thermo Scientific). Blots were washed and reprobed with an anti-actin antibody (Santa Cruz) and developed in an identical manner to assess β-actin protein levels to ensure even loading.

### Cx25 knockdown

Cx25 inhibition was accomplished by shRNA in Jurkat cells. In short, plasmid DNA was isolated from glycerol stocks of bacteria containing shRNA plasmid DNA (Sigma MISSION shRNA) specific to Cx25 (TRCN0000222613 (KD13) and TRCN0000074136 (KD 36)) or a nontargeting control (NT) and used to produce virus. These shRNA plasmids were chosen from a group of five total plasmids as they were shown to reliably reduce the levels of Cx25, as demonstrated by immunoblotting and qRT-PCR. Additional shRNA constructs against Cx43 were also purified from bacterial glycerol stocks (TRCN0000059775 (KD 75) and TRCN0000059776 (KD 76) and were likewise used to inhibit Cx43 expression in Jurkat cells as demonstrated by qRT-PCR. Bacterial stocks were expanded, and plasmid DNA was purified using PureLink HiPure Plasmid Maxiprep Kit (Life Technologies) according to manufacturer's instructions. 293T cells were cotransfected with the packaging vectors pMD2.G and psPAX2 (Addgene) and shRNA constructs. Media on the 293T cell cultures were changed 18 hr after transfection, and viral supernatants were collected 24, 36, and 48 hr later and filtered for immediate use or concentrated with polyethylene glycol precipitation and stored at −80°C for future use. Jurkat cells and MV4-11 cells were subsequently infected with lentiviral particles using the Sigma MISSION shRNA Spinoculation protocol, and transduced cells were selected after incubation with puromycin.

### Cx25 immunofluorescence

To visualize Cx25 expression and localization in leukemia cells, Jurkat and THP1 cells were centrifuged onto glass slides using a Cytospin protocol. Cells were fixed with 4% PFA for 10 minutes and washed three times with 0.1% PBST (PBS/Triton X-100) for 5 minutes. After washing, cells were blocked with 10% normal goat serum for 30 minutes. Rabbit polyclonal anti-Cx25 (Sigma, SAB4501629, 1:100) was used to stain cells overnight at 4°C. The following day, cells were washed 3 times with 0.1% PBST, and the appropriate secondary antibody was applied for 2 hr at room temperature (goat anti-rabbit IgG Alexa Flour 488, Life Technologies, 1:2000). After secondary antibody incubation, cells were washed three times with 0.1% PBST and counterstained with 4′,6-diamidino-2-phenylindole (DAPI) for 5 minutes. Afterwards, cells were washed three additional times with 0.1% PBST, and coverslips were mounted using FluorSave Reagent (VWR International). Cells were imaged with a Leica TCS SP8 confocal microscope, and images were prepared in figure form using Adobe Photoshop.

### Bioinformatics analysis

RNA sequencing for all connexin subunits was evaluated using the Cancer Genome Atlas [20]. Expression of Cx25, Cx31.9, Cx40, and Cx43 were interrogated in the Beroukhim et al., Nature 2010 dataset [21] in Oncomine (www.oncomine.org).

### Statistical analysis

Data are represented as mean values +/− standard deviation. Statistical significance was analyzed using one-way ANOVA, with p values less than 0.05 considered statistically significant.

## SUPPLEMENTARY MATERIAL FIGURES AND TABLE



## References

[1] Howlader N, Noone AM, Krapcho M, Garshell J, Neyman N, Altekruse SF, Kosary CL, Yu M, Ruhl J, Tatalovich Z, Cho H, Mariotto A, Lewis DR, Chen HS, Feuer EJ, Cronin KA SEER Cancer Statistics Review, 1975-2010.

[2] Fernandez HF (2010). New Trends in the Standard of Care for Initial Therapy of Acute Myeloid Leukemia. ASH Education Program Book.

[3] Xie Y, Davies SM, Xiang Y, Robison LL, Ross JA (2003). Trends in leukemia incidence and survival in the United States (1973–1998). Cancer.

[4] Colmone A, Amorim M, Pontier AL, Wang S, Jablonski E, Sipkins DA (2008). Leukemic Cells Create Bone Marrow Niches That Disrupt the Behavior of Normal Hematopoietic Progenitor Cells. Science.

[5] Gillette JM, Lippincott-Schwartz J (2009). Hematopoietic progenitor cells regulate their niche microenvironment through a novel mechanism of cell-cell communication. Communicative & Integrative Biology.

[6] Zhang X, Liu Y, Si Y-j, Chen X-h, Li Z-j, Gao L, Gao L, Zhang C (2012). Effect of Cx43 gene-modified leukemic bone marrow stromal cells on the regulation of Jurkat cell line in vitro. Leukemia Research.

[7] Umezu T, Ohyashiki K, Kuroda M, Ohyashiki JH (2013). Leukemia cell to endothelial cell communication via exosomal miRNAs. Oncogene.

[8] Warner AE, Guthrie SC, Gilula NB (1984). Antibodies to gap-junctional protein selectively disrupt junctional communication in the early amphibian embryo. Nature.

[9] Beauchamp P, Desplantez T, McCain ML, Li W, Asimaki A, Rigoli G, Parker KK, Saffitz JE, Kleber AG (2012). Electrical Coupling and Propagation in Engineered Ventricular Myocardium With Heterogeneous Expression of Connexin43. Circulation Research.

[10] Allison DW, Ohran AJ, Stobbs SH, Mameli M, Valenzuela CF, Sudweeks SN, Ray AP, Henriksen SJ, Steffensen SC (2006). Connexin-36 gap junctions mediate electrical coupling between ventral tegmental area GABA neurons. Synapse.

[11] Gonzalez-Nieto D, Li L, Kohler A, Ghiaur G, Ishikawa E, Sengupta A, Madhu M, Arnett JL, Santho RA, Dunn SK, Fishman GI, Gutstein DE, Civitelli R, Barrio LC, Gunzer M, Cancelas JA (2012). Connexin-43 in the osteogenic BM niche regulates its cellular composition and the bidirectional traffic of hematopoietic stem cells and progenitors. Blood.

[12] Kamiya K, Yum SW, Kurebayashi N, Muraki M, Ogawa K, Karasawa K, Miwa A, Guo X, Gotoh S, Sugitani Y, Yamanaka H, Ito-Kawashima S, Iizuka T, Sakurai T, Noda T, Minowa O (2014). Assembly of the cochlear gap junction macromolecular complex requires connexin 26. The Journal of Clinical Investigation.

[13] Stains JP, Civitelli R (2005). Gap junctions in skeletal development and function. Biochimica et Biophysica Acta (BBA) - Biomembranes.

[14] Kihara AH, Santos TO, Osuna-Melo EJ, Paschon V, Vidal KSM, Akamine PS, Castro LM, Resende RR, Hamassaki DE, Britto LRG (2010). Connexin-mediated communication controls cell proliferation and is essential in retinal histogenesis. International Journal of Developmental Neuroscience.

[15] Söhl G, Willecke K (2004). Gap junctions and the connexin protein family. Cardiovascular Research.

[16] Hervé J-C, Derangeon M (2013). Gap-junction-mediated cell-to-cell communication. Cell Tissue Res.

[17] Goldberg GS, Lampe PD, Nicholson BJ (1999). Selective transfer of endogenous metabolites through gap junctions composed of different connexins. Nat Cell Biol.

[18] Ng KP, Ebrahem Q, Negrotto S, Mahfouz RZ, Link KA, Hu Z, Gu X, Advani A, Kalaycio M, Sobecks R, Sekeres M, Copelan E, Radivoyevitch T, Maciejewski J, Mulloy JC, Saunthararajah Y (2011). p53 Independent epigenetic-differentiation treatment in xenotransplant models of acute myeloid leukemia. Leukemia.

[19] Zhang A, Hitomi M, Bar-Shain N, Dalimov Z, Ellis L, Velpula KK, Fraizer GC, Gourdie RG, Lathia JD (2015). Connexin 43 expression is associated with increased malignancy in prostate cancer cell lines and functions to promote migration.

[20] (2013). Genomic Epigenomic Landscapes of Adult De Novo Acute Myeloid Leukemia. New England Journal of Medicine.

[21] Beroukhim R, Mermel CH, Porter D, Wei G, Raychaudhuri S, Donovan J, Barretina J, Boehm JS, Dobson J, Urashima M, Mc Henry KT, Pinchback RM, Ligon AH, Cho Y-J, Haery L, Greulich H (2010). The landscape of somatic copy-number alteration across human cancers. Nature.

[22] Drug information online. Drugs.com (2011). Carbenoxolone.

[23] Nahab F, Wittevrongel L, Ippolito D, Toro C, Grimes G, Starling J, Potti G, Haubenberger D, Bowen D, Buchwald P, Dong C, Kalowitz D, Hallett M (2011). An Open-Label, Single-Dose, Crossover Study of the Pharmacokinetics and Metabolism of Two Oral Formulations of 1-Octanol in Patients with Essential Tremor. Neurotherapeutics.

[24] Negrotto S, Ng KP, Jankowska AM, Bodo J, Gopalan B, Guinta K, Mulloy JC, Hsi E, Maciejewski J, Saunthararajah Y (2012). CpG methylation patterns and decitabine treatment response in acute myeloid leukemia cells and normal hematopoietic precursors. Leukemia.

[25] Marusyk A, Almendro V, Polyak K (2012). Intra-tumour heterogeneity: a looking glass for cancer?. Nat Rev Cancer.

[26] Boyerinas B, Zafrir M, Yesilkanal AE, Price TT, Hyjek EM, Sipkins DA (2013). Adhesion to osteopontin in the bone marrow niche regulates lymphoblastic leukemia cell dormancy. Blood.

[27] Upham BL, Trosko JE (2009). Oxidative-Dependent Integration of Signal Transduction with Intercellular Gap Junctional Communication in the Control of Gene Expression. Antioxidants & Redox Signaling.

[28] Taniguchi Ishikawa E, Gonzalez-Nieto D, Ghiaur G, Dunn SK, Ficker AM, Murali B, Madhu M, Gutstein DE, Fishman GI, Barrio LC, Cancelas JA (2012). Connexin-43 prevents hematopoietic stem cell senescence through transfer of reactive oxygen species to bone marrow stromal cells. Proceedings of the National Academy of Sciences.

[29] Trosko JE, Ruch RJ (2002). Gap junctions as targets for cancer chemoprevention and chemotherapy. Current Drug Targets.

[30] Hitomi M, Deleyrolle Loic P, Mulkearns-Hubert Erin E, Jarrar A, Li M, Sinyuk M, Otvos B, Brunet S, Flavahan William A, Hubert Christopher G, Goan W, Hale James S, Alvarado Alvaro G, Zhang A, Rohaus M, Oli M (2015). Differential Connexin Function Enhances Self-Renewal in Glioblastoma. Cell Reports.

[31] Barr TP, Albrecht PJ, Hou Q, Mongin AA, Strichartz GR, Rice FL (2013). Air-Stimulated ATP Release from Keratinocytes Occurs through Connexin Hemichannels. PLoS ONE.

[32] Chen G, Park C-K, Xie R-G, Berta T, Nedergaard M, Ji R-R (2014). Connexin-43 induces chemokine release from spinal cord astrocytes to maintain late-phase neuropathic pain in mice. Brain.

[33] Véliz LP, González FG, Duling BR, Sáez JC, Boric MP (2008). Functional role of gap junctions in cytokine-induced leukocyte adhesion to endothelium in vivo. American Journal of Physiology - Heart and Circulatory Physiology.

[34] Chadjichristos CE, Scheckenbach KEL, van Veen TAB, Richani Sarieddine MZ, de Wit C, Yang Z, Roth I, Bacchetta M, Viswambharan H, Foglia B, Dudez T, van Kempen MJA, Coenjaerts FEJ, Miquerol L, Deutsch U, Jongsma HJ (2010). Endothelial-Specific Deletion of Connexin40 Promotes Atherosclerosis by Increasing CD73-Dependent Leukocyte Adhesion. Circulation.

[35] Mancuso M, Pasquali E, Leonardi S, Rebessi S, Tanori M, Giardullo P, Borra F, Pazzaglia S, Naus CC, Di Majo V, Saran A (2011). Role of connexin43 and ATP in long-range bystander radiation damage and oncogenesis in vivo. Oncogene.

[36] Naus CC, Laird DW (2010). Implications and challenges of connexin connections to cancer. Nat Rev Cancer.

