# Supplementary material for: Cx25 contributes to leukemia cell communication and chemosensitivity
Source: Oncotarget. 2015 Aug 19;6(31):31508–21. doi: 10.18632/oncotarget.5226 (PMC4741621; doi:10.18632/oncotarget.5226)
Supplement: Supplementary file 1 [file oncotarget-06-31508-s001.pdf]

Cx25 contributes to leukemia cell communication and chemosensitivity

Supplementary Material

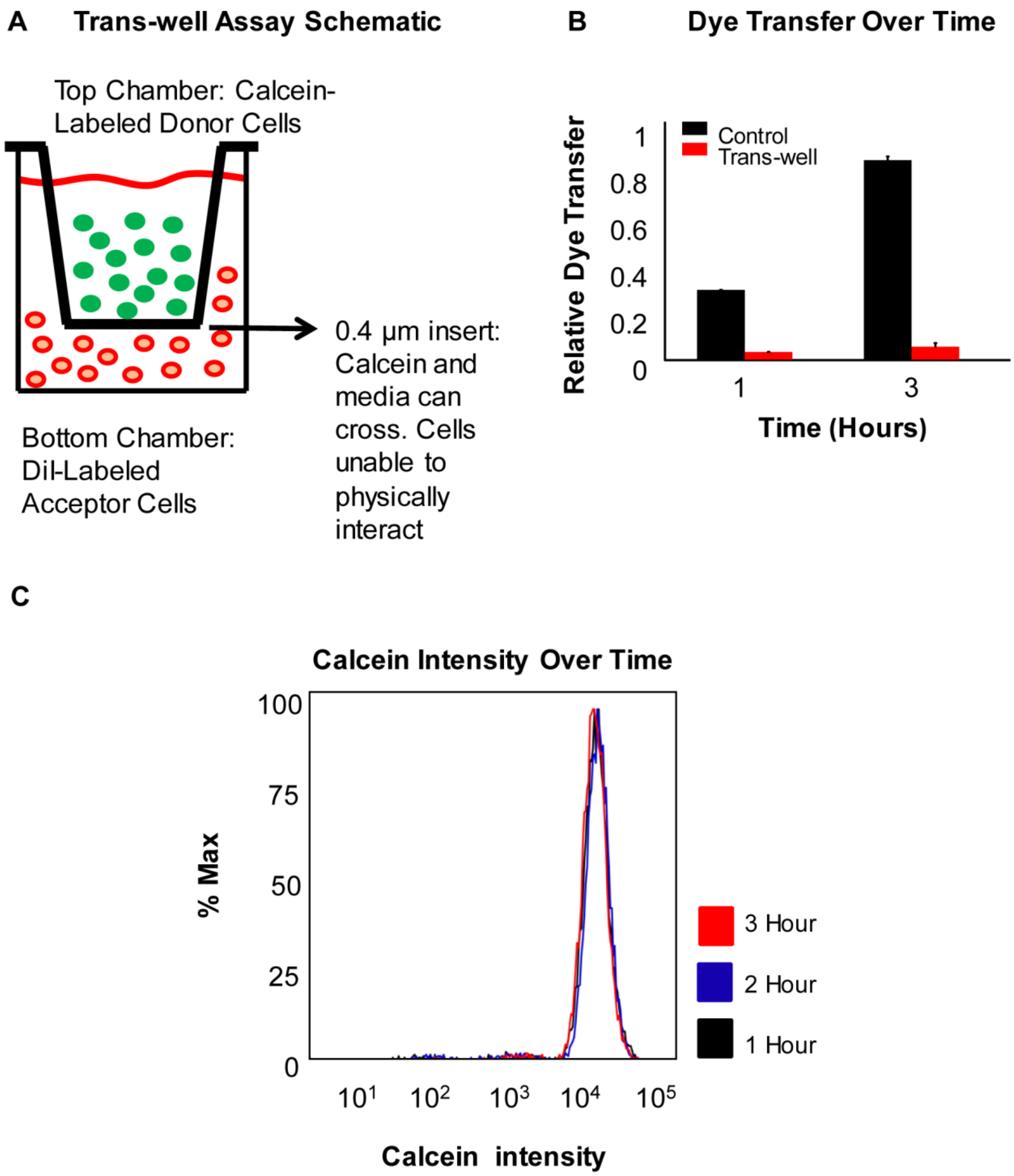

Supplemental Figure 1. Direct Physical Contact between Cells is Necessary for Gap Junction-Mediated Communication. (A) Schematic detailing how the Transwell assay was performed. **(B)** Dye transfer was measured over time between two groups of cells that were allowed to physically interact and between those that were kept separate with a Transwell insert. **(C)** Calcein fluorescence was measured by flow cytometry over 1, 2, and 3 hr and showed no reduction in intensity over time, indicating that no Calcein was leaking from cells.

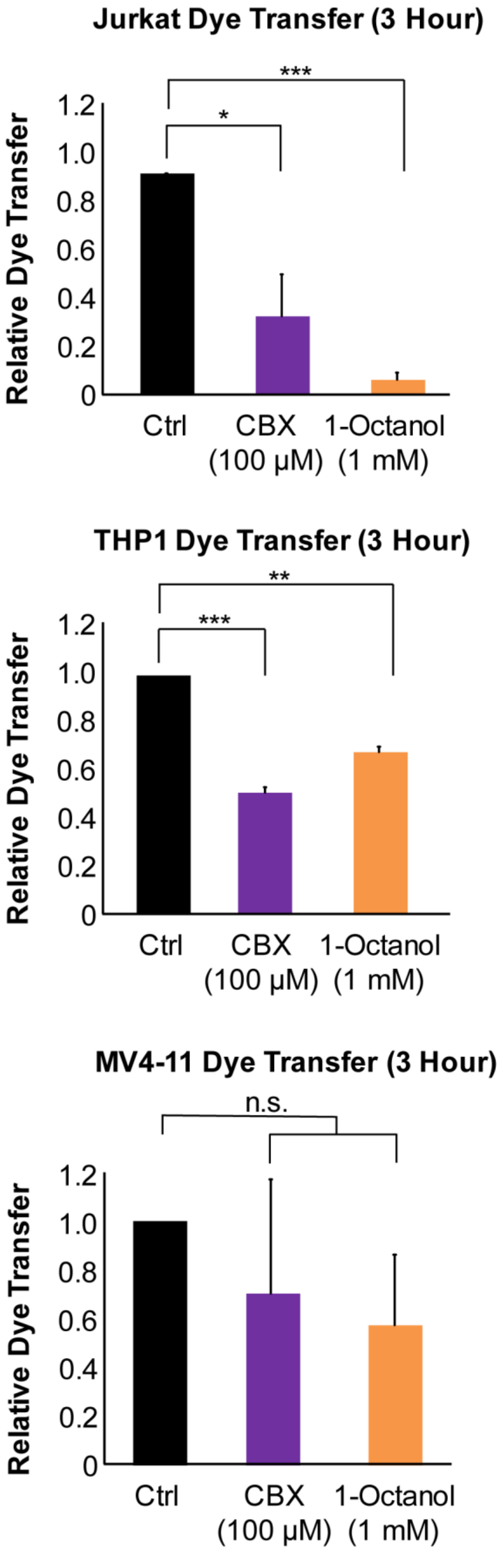

Supplemental Figure 2. Gap Junction Inhibition is Sufficient to Decrease Leukemia Cell Communication. After 3 hr of incubation with 100 μM of CBX or 1 mM 1-octanol, both Jurkat and THP1 cells showed decreased dye transfer by flow cytometry analysis. However, after 3 hr of co-incubation, MV4-11 cells did not show the same decrease in dye transfer.

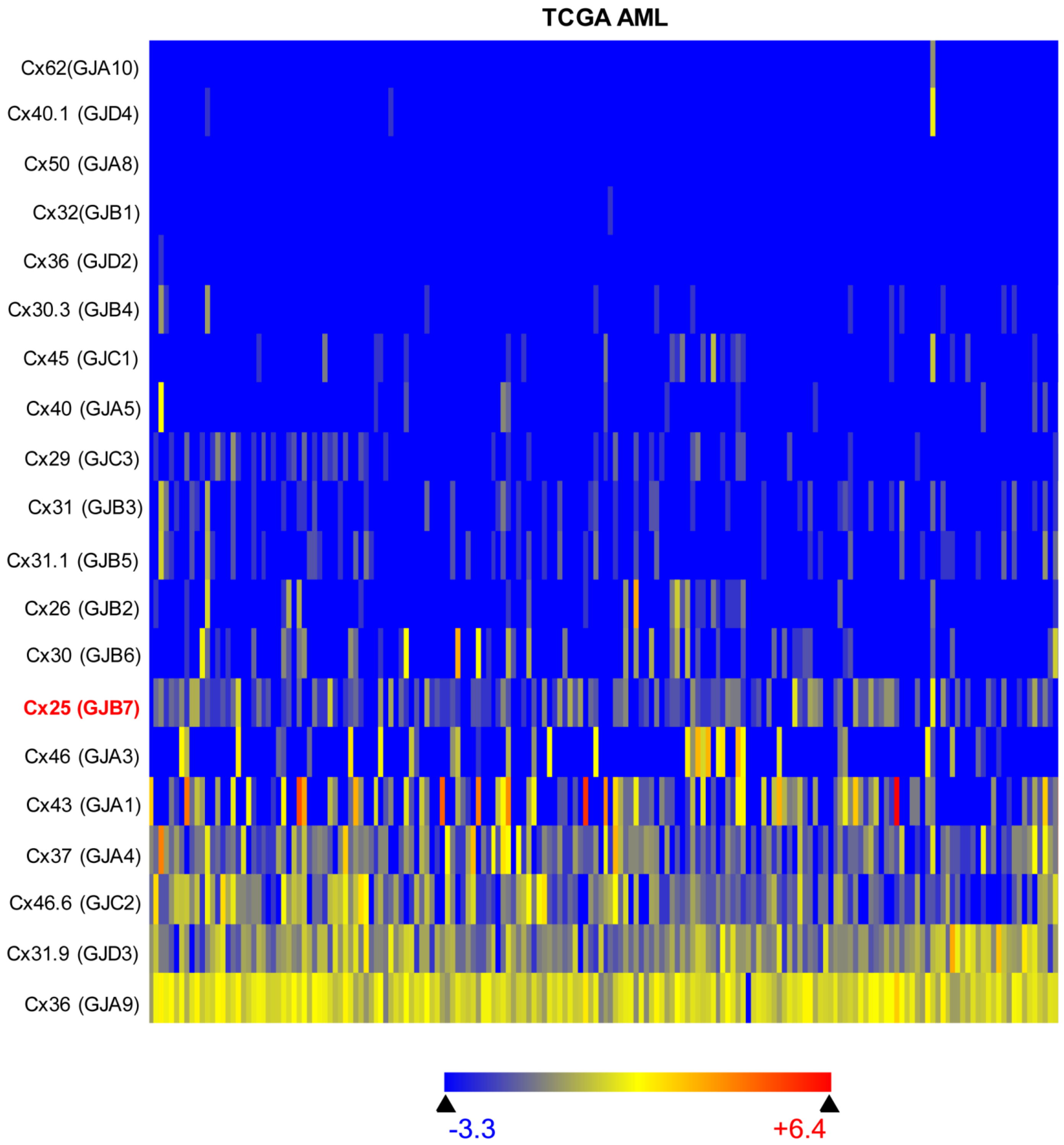

Supplemental Figure 3. The Cancer Genome Atlas (TCGA) AML RNA-Seq Analysis of Connexin Expression. Gene expression measured by RNA sequencing (TCGA AML, Runx1 wild-type n=179). Heat map of gene expression values generated by ArrayStar software.

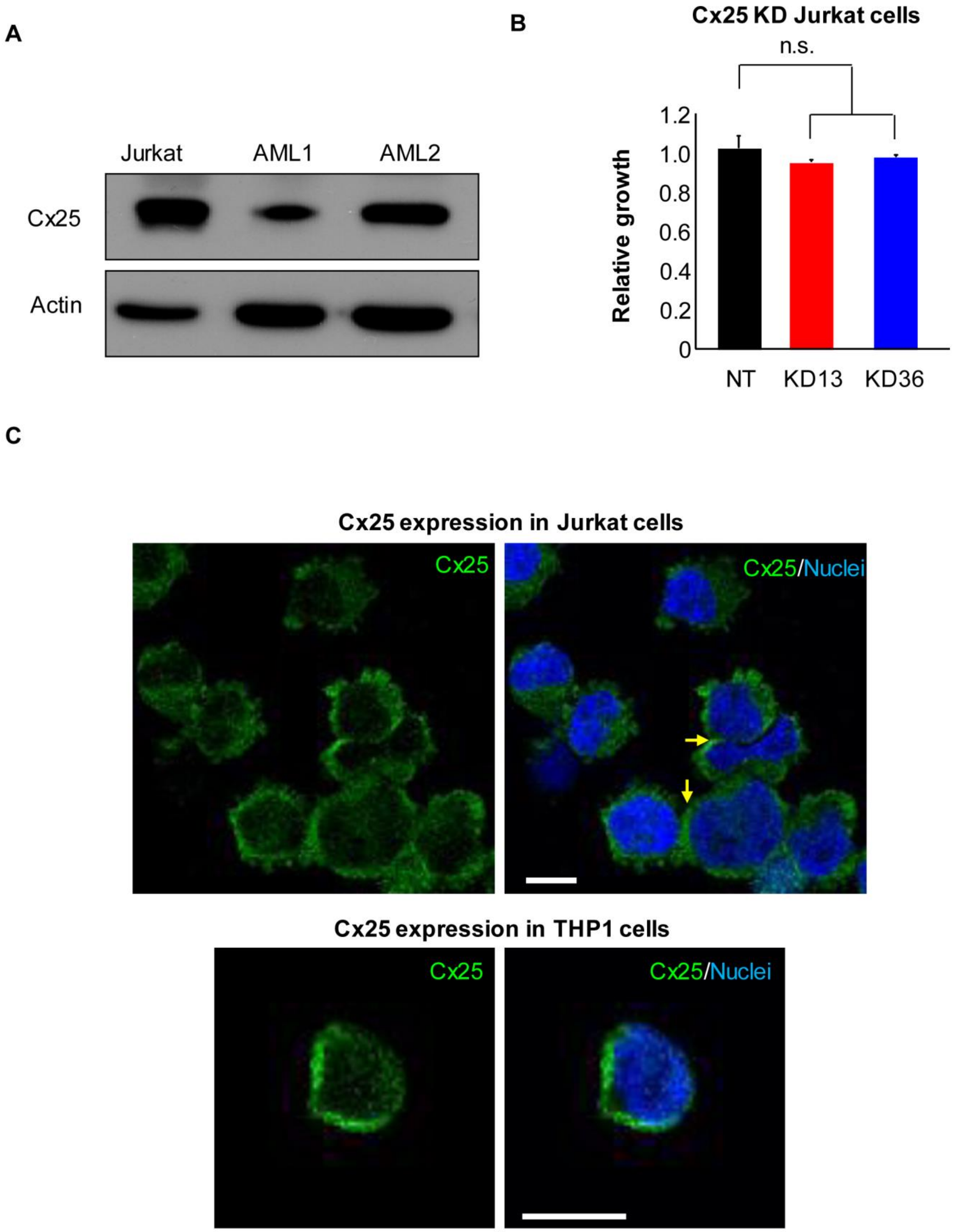

Supplemental Figure 4. Cx25 KD Does Not Affect Proliferation in Jurkat Cells. (A) Cx25 expression was observed in two primary AML lines as measured by immunoblotting. **(B)** Following Cx25 knockdown, Jurkat cell proliferation was measured, and relative growth was found to not significantly change between the two shRNA constructs and the NT control. **(C)** Micrographs of Jurkat cells and THP1 cells prepared by Cytospin and stained with an anti-Cx25 antibody (green). Yellow arrows indicate cell-cell contact, scale bar represents 10 microns.

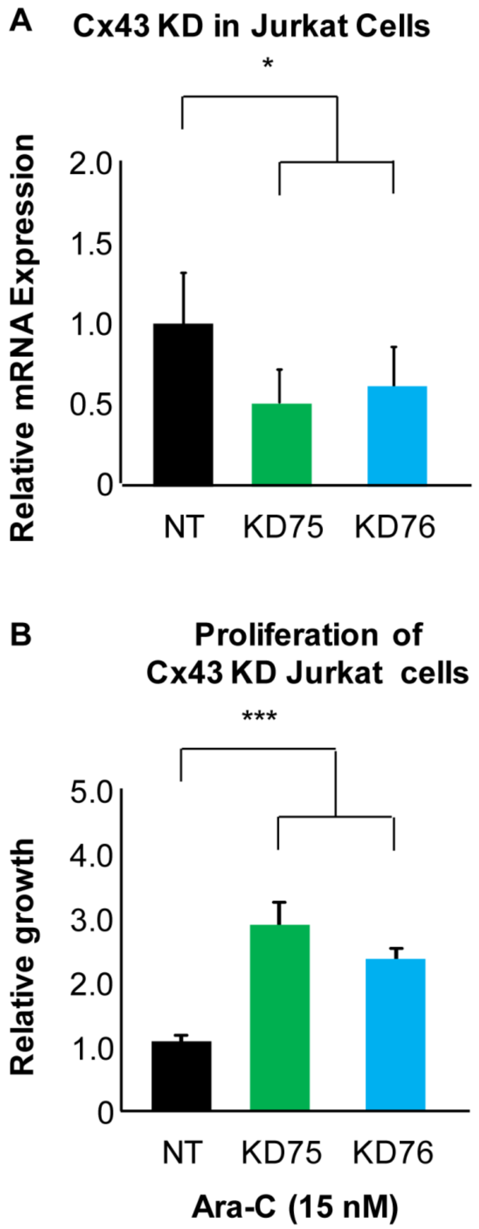

Supplemental Figure 5. Cx43 KD Does Not Chemosensitize Jurkat Cells. (A) Cx43 KD was validated with qRT-PCR using two different constructs (KD 75 and KD 76). **(B)** After Cx43 KD, Jurkat cells were not sensitized to treatment with 15 nM Ara-C.

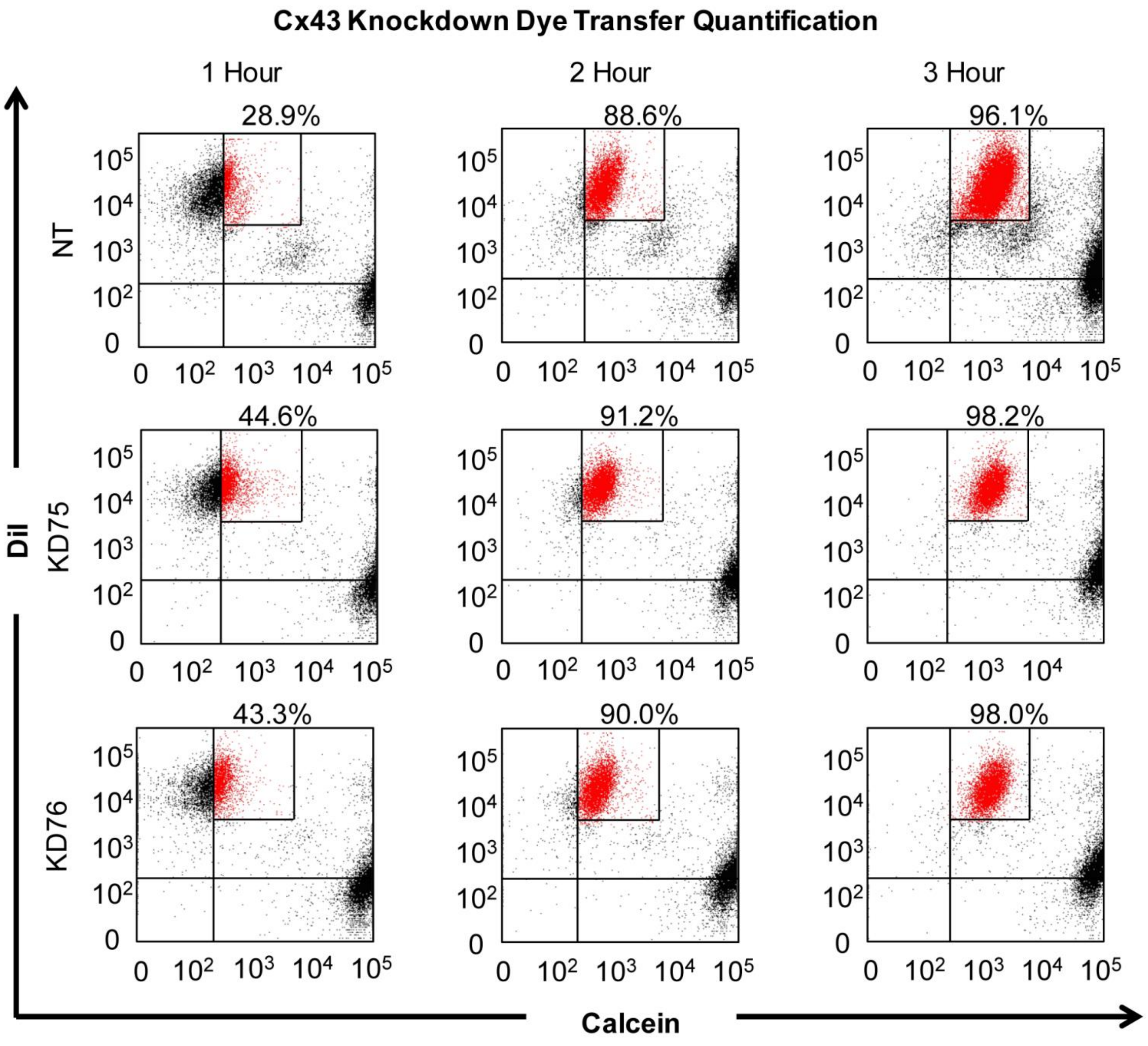

Supplemental Figure 6. Cx43 KD Does Not Affect Jurkat Cell Dye Transfer. Jurkat cell dye transfer was not affected by Cx43 KD compared with NT controls at any time point.

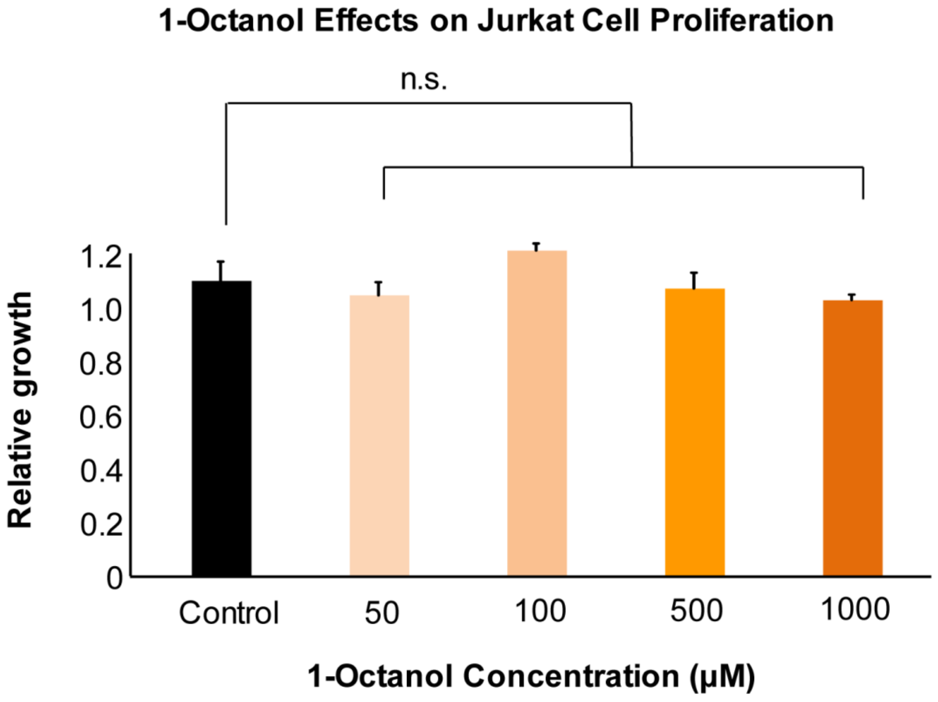

Supplemental Figure 7. 1-Octanol is Not Effective at Reducing Leukemia Cell Proliferation. Jurkat cells were incubated with 1-octanol at concentrations ranging from 50 μM to 1 mM. No concentration of 1-octanol was sufficient to reduce Jurkat cell proliferation.

Supplemental Table 1. qRT-PCR Fold Change Values. The fold change values used to generate the heat map of connexin expression are detailed to demonstrate the connexins that were upregulated or downregulated in leukemia vs. normal hematopoietic cells.

	HSCs	Jurkat	MV4	THP1	AML1	AML2
Cx25	12.54	10.41	10.27	8.26	9.96	10.83
Cx40	14.46	12.76	11.67	11.44	11.94	11.85
Cx31.9	9.21	9.73	10.22	9.10	7.13	6.29
Cx62	7.83	7.95	8.98	7.75	5.36	5.15
Cx45	8.85	5.63	14.17	7.76	12.96	13.81
Cx46	15.85	15.80	0.00	21.14	18.41	15.23
Cx30.3	14.84	13.41	13.93	12.55	14.36	16.50
Cx26	9.69	10.38	ND	ND	9.91	10.83
Cx36	16.53	17.92	16.79	17.00	17.34	17.89
Cx37	10.88	12.43	12.00	14.72	10.81	12.19
Cx40.1	12.1	11.41	13.00	15.72	12.55	14.61
Cx43	9.55	12.33	15.66	15.96	9.56	10.77
Cx47	9.48	9.66	14.06	13.70	11.6	11.52
Cx50	12.93	13.93	14.78	ND	15.71	15.27
Cx59	11.51	13.39	14.66	12.70	13.96	17.01
Cx31	8.74	10.01	10.07	9.09	11.52	12.56
Cx31.1	9.01	10.59	12.58	11.90	13.1	13.77
Cx32	7.3	9.08	10.81	9.89	10.5	11.79
Cx30	4.51	8.91	10.59	9.41	12.3	9.90
Cx30.2	12.11	14.53	17.51	14.57	16.77	17.09
